# Synthesis and Antiproliferative Activity of Some Novel Triazole Derivatives from Dehydroabietic Acid

**DOI:** 10.3390/molecules19022523

**Published:** 2014-02-21

**Authors:** Mariano Walter Pertino, Valery Verdugo, Cristina Theoduloz, Guillermo Schmeda-Hirschmann

**Affiliations:** 1 Instituto de Química de Recursos Naturales, Universidad de Talca, Casilla 747, Talca, Chile; 2 Facultad de Ciencias de la Salud, Universidad de Talca, Casilla 747, Talca, Chile

**Keywords:** dehydroabietic acid, click chemistry, antiproliferative activity

## Abstract

Dehydroabietic acid (DHA) is a naturally occurring diterpene with different and relevant biological activities. Previous studies have shown that some DHA derivatives display antiproliferative activity. However, the reported compounds did not include triazole derivatives. Starting from DHA (8,11,13-abietatrien-18-oic acid), and its alcohol dehydroabietinol (8,11,13-abietatrien-18-ol), four alkyl esters were prepared. The alkyl terpenes were treated with different aromatic azides to synthesize hybrid compounds using click chemistry. Some 16 new DHA hybrids were thus synthesized and their structures were confirmed by spectroscopic and spectrometric means. The antiproliferative activity of the new compounds was assessed towards human cell lines, namely normal lung fibroblasts (MRC-5), gastric epithelial adenocarcinoma (AGS), lung cancer (SK-MES-1) and bladder carcinoma (J82) cells. Better antiproliferative effect was found for compound **5**, with an IC_50_ of 6.1 μM and selectivity on SK-MES-1 cells. Under the same experimental conditions, the IC_50_ of etoposide, was 1.83 µM.

## 1. Introduction

Synthesis or modification of known anticancer drugs is an important aspect of research. However, to date a vast amount of synthetic work has contributed to relatively small improvements over the prototype drugs [1]. There is a continuous need for new templates to be used in the design of potential chemotherapeutic agents. Dehydroabietic acid (DHA), a naturally occurring resin diterpene, and its derivatives, exhibit a broad spectrum of biological effects, including antiulcer [2,3], antimicrobial [4], antiviral [5], cytotoxic [3,6] and antitumor activities [7]. A recent patent on the use of abietic acid and derivatives as antitumor agents has been published [8].

The search for drugs based on natural products often has the problem of involving slow and complex synthetic pathways [9]. Click chemistry offers an alternative approach because it is a simple and selective reaction between azides and alkynes to generate triazoles. The 1,4-disubstituted triazole moiety is well known in medicinal chemistry. Before the discovery of the click chemistry reaction, more than 7,000 compound bearing a 1,4-disubstituted 1*H*-1,2,3-triazole had been reported. The triazole unit is stable to oxidation and reduction, and is quite resistant to metabolic degradation [10]. In the design of novel prototypes for new antitumor drugs, triazole rings became increasingly relevant in studies published in 2013 [11,12,13]. Stefely* et al.* [14] reported antiproliferative triazoles with an IC_50_ of 46 nM against MCF-7 human breast tumor cells. Moreover, the anti-angiogenic drug, 1,2,3-triazole carboxyamidotriazole is being investigated in clinical trials as a potential anticancer drug [15,16].

Recently, thiourea α-aminophosphonate derivatives were attached to DHA, searching for antitumor agents [7]. The authors measured cytotoxicity as the protocols for cell toxicity (cytotoxicity) and antiproliferative effects are different. Moreover, antitumoral activity requires animal experiments. The compounds showed moderate to high toxicity on NCI-H460, A549, HepG2 and SKOV3 cells. Some of the compounds were more toxic than the commercial anticancer drug 5-fluorouracil.

The click chemistry approach to design new antiproliferative/anticancer agents has acquired relevance in the last few years. In previous works, we reported the antiproliferative activity of dimeric labdane diterpenes, some of them synthesized using click chemistry [17] and 1,2,3-triazole-substituted oleanolic acid derivatives [18]. Kádár* et al.* [19] synthesized steroidal triazole derivatives to investigate their antiproliferative effects. The most promising compounds were studied to evaluate its mechanism of action, where apoptosis plays an important role. Moreover Li* et al.* [20] explored the addition of triazole rings to naphthalimide to obtain hybrids with cytotoxic activity. One of the new hybrids was more cytotoxic that the reference compound amonafide against the MCF-7, Hela and liver cancer 7721 cells, with IC_50_ values in the range of 0.323–1.02 µM. Under the same experimental conditions, the IC_50_ of amonafide was in the range of 1.68–4.27 µM. The results points out the potential of click chemistry to design new bioactive agents. However, other tools have to be included for a more rational and effective drug design, including molecular modeling and receptor studies. The aim of this study was to synthesize a series of hybrid molecules combining DHA with different aromatic-substituted triazoles and to assess the antiproliferative effect of the new compounds in a panel of human tumor cell lines. Recently, one of the alkyl esters prepared in this work (DHA propargyl ester) was incorporated to caprolactone polymer (polycaprolactone, PCL) by click chemistry reaction. The integration of DHA to PLC increases the glass-transition temperature of PCL and does excellent hydrophobicity and good degradability to the polymer [21].

## 2. Results and Discussion

**Scheme 1:**
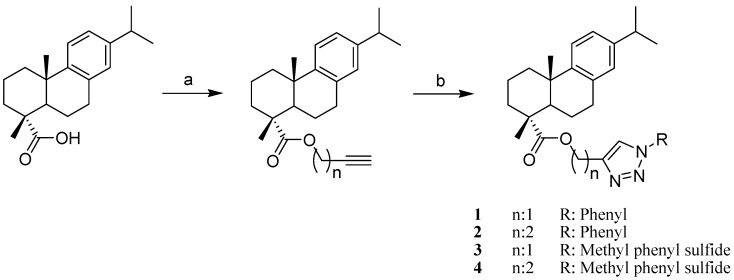
Preparation of DHA derivatives **1**–**4**.

**Scheme 2:**
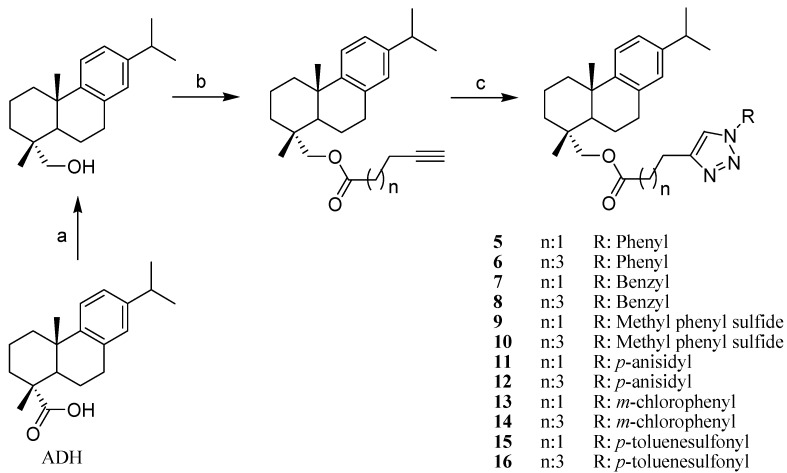
Preparation of dehydroabietinol derivatives **5**–**16**.

Starting from DHA and its alcohol, four alkyl esters were prepared and then treated with different aromatic azides using click chemistry to produce 16 hybrid abietane-triazole compounds (Scheme 1 and Scheme 2). The new compounds were obtained in moderate to good yields. The method proved to be suitable to obtain series of derivatives from diterpenes and can be applied to other natural products to increase structural diversity. Compounds **1**–**16** are described for the first time. The structures of all the compounds were confirmed by spectroscopic and spectrometric means. 

*Reagents and conditions*: (a) appropriate alkyne alcohol, DCC, DMAP, CH_2_Cl_2_, 65%–77%; (b) appropriate aromatic azide, CuSO_4_·5H_2_O, sodium ascorbate, *t*-BuOH/H_2_O 1:1, 59%–70%.

*Reagents and conditions*: (a) LiAlH_4_, THF, 80 °C, 86%; (b) appropriate alkyne acid, DCC, DMAP, CH_2_Cl_2_, 60%–81%; (c) appropriate aromatic azide, CuSO_4_·5H_2_O, sodium ascorbate, *t*-BuOH/H_2_O 1:1, 54%–89%.

The compounds were then assessed for antiproliferative activity towards the following human cell lines: normal lung fibroblasts (MRC-5), gastric epithelial adenocarcinoma (AGS), lung cancer (SK-MES-1) and bladder carcinoma (J82) cells. IC_50_ values > 100 µM were considered inactive (Table 1).

**Table 1:** Antiproliferative activity of dehydroabietic acid derivatives **1**–**16** against MRC-5 normal fibroblasts and selected tumor cell lines. *^a^*

Compound	(IC_50 _± SD, µM) *^b^*			
	MRC-5	AGS	SK-MES-1	J82
**1**	85.8 ± 4.6	79.9 ± 4.8	77.3 ± 4.7	46.3 ± 2.3
**2**	>100	90.2 ± 5.4	>100	76.3 ± 5.9
**3**	>100	94.4 ± 5.4	>100	>100
**4**	50.2 ± 3.1	66.0 ± 4.4	28.9 ± 2.1	28.1 ± 1.7
**5**	17.1 ± 0.6	44.5 ± 1.9	6.1 ± 0.3	83.3 ± 3.3
**6**	>100	>100	>100	>100
**7**	>100	36.7 ± 1.5	39.7 ± 2.7	25.6 ± 1.9
**8**	>100	35.9 ± 1.7	37.0 ± 2.6	26.8 ± 1.8
**9**	73.3 ± 3.7	47.7 ± 2.9	43.6 ± 2.9	49.9 ± 2.9
**10**	>100	58.9 ± 3.4	42.7 ± 2.9	33.9 ± 1.7
**11**	>100	>100	>100	>100
**12**	>100	>100	>100	>100
**13**	>100	>100	>100	>100
**14**	>100	>100	>100	>100
**15**	82.9 ± 4.5	74.9 ± 3.7	84.4 ± 5.1	>100
**16**	68.8 ± 4.5	56.9 ± 3.3	74.2 ± 5.4	75.0 ± 6.1
Etoposide *^c^*	0.33 ± 0.02	0.58 ± 0.02	1.83 ± 0.09	3.49 ± 0.16

*^a^* Cell lines: normal lung fibroblasts (MRC-5), gastric epithelial adenocarcinoma (AGS), lung cancer (SK-MES-1) and bladder carcinoma (J82) cells; *^b^* Results are expressed as mean values ± SD. Each concentration was tested in sextuplicate together with the control and repeated two times in separate experiments; *^c^* Reference compound.

Among the triazoles prepared with the diterpene acid, the derivatives **1** and **4** presented antiproliferative activity towards all of the selected cell lines. The effect, however, is low. Compounds **2** and **3**, differing in the number of CH_2_ groups of the linker or in the aromatic moiety were inactive against MRC-5 and SK-MES1cells. When comparing the pairs **1**–**2** and **3**–**4** bearing one or two CH_2_ units as linkers, the effect of **1** was higher than that of **2**, suggesting that a longer chain decreases activity when R is a phenyl group. However, an opposite effect was found for the methyl phenyl sulfide derivatives. The best compound of this group was the derivative **4**, with a (CH_2_)_2_ linker and a methyl phenyl sulfide substituent in the triazole ring. More derivatives with longer linker chains need to be prepared to have a clearer picture of the effect of this structural feature on the antiproliferative effect.

The compounds **5**–**16**, were prepared with the diterpene alcohol. Among them, the compounds **11**–**14** were inactive. All of them contain electron donors such as a methoxy group or a chlorine atom in the aromatic ring. Compounds **7** and **8** showed similar activity against AGS, SK-MES-1 and J82 cells and were inactive against fibroblasts, showing a good selectivity. This fact suggests that the length of the CH_2_ linker from 1 to 3 units do not have a relevant influence in the antiproliferative activity. The same trend was observed for the products **9** and **10**, differing in the linker. For the pair **15** and **16**, the best effect was observed for compound **16** with a longer linker. Overall the activity for the compounds should be considered as moderate. 

The hybrid compound **5** with a phenyl ring was the most active product designed in this study, presenting IC_50_ values of 17.1 and 6.1 µM against MRC-5 and SK-MES-1 cells. The compound differs from **1** in the identity of the diterpene moiety and in the nature of the linker. In the slightly active compound **1**, the diterpene is a carboxylic acid, while in **5**, the terpene moiety is an alcohol. The effect of compound **5** was associated with some selectivity against the SK-MES-1 cells. The SK-MES-1 are lung cancer cells (HTB-58) derived from metastatic site. The selectivity of compound **5** towards SK-MES-1 cells suggest potential of the compound against lung cancer cells and encourages further studies using* in vivo* models. The difference in effect of compound **5** and **7** against SK-MES-1 cells can be related to the differences in the phenyl* vs.* benzyl ring in the triazole moiety. However, the effect is inverse for J82 bladder carcinoma cells. In summary, the new compounds present different degrees of antiproliferative activity, with derivative **5** displaying relevant effect against lung cancer cell line SK-MES-1, with an IC_50_ value of 6.1 µM. Under the same experimental conditions, the IC_50_ value of etoposide was 1.83 µM. 

## 3. Experimental

### 3.1. General Procedures

Melting points were determined on a Koffler hot stage apparatus (Electrothermal 9100, Dubuque, IA, USA) and were uncorrected. Optical rotations were measured on a Jasco DIP 370 (Jasco Analytical Instruments, Easton, MD, USA) polarimeter in CHCl_3_ at 20 °C. IR spectra were recorded on a Nicolet Nexus 470 FT-IR instrument (Thermo Electron Corporation, Whaltham, MA, USA). The NMR spectra were recorded on a Bruker Avance 400 (Bruker, Rheinstetten, Germany) spectrometer at 400 MHz for ^1^H and 100 MHz for ^13^C in CDCl_3_. Chemical shifts are given in ppm with TMS as the internal standard. High-resolution mass spectra were measured on a VG Micromass ZAB-2F at 70 eV (Varian Inc., Palo Alto, CA, USA). Merck silica gel (0.063–0.2) was used for column chromatography (CC), pre-coated Si gel plates (Merck, Kieselgel 60 F_254_, 0.25 mm) were used for TLC analysis. TLC spots were visualized by spraying the chromatograms with *p*-anisaldehyde–ethanol–acetic acid–H_2_SO_4_ (2:170:20:10 v/v) and heating at 110 °C for 3 min. Reagents: *N*,*N*-Dicyclohexylcarbodiimide (DCC) and dimethylaminopyridine (DMAP) were from Merck (Schuchardt, Germany). Propargyl alcohol, 3-butyn-1-ol, 4-pentynoic acid and 6-heptynoic acid were from Aldrich (Steinheim, Germany). Aromatic azides were from Aldrich. Copper (II) sulphatepentahydrate was from Aldrich (St. Louis, MO, USA) and sodium ascorbate was from Sigma (St. Louis, MO, USA).

### 3.2. Obtention of Dehydroabietic Acid Derivatives

Dehydroabietic acid (DHA) was obtained from commercial rosin as described previously [22]. Some 300 g of rosin with 300 mg of 5% Pd/C were heating at 280 °C under a constant flow of N_2_ for 2 h. The generated crude product was purified by CC on silica gel with petroleum ether–ethyl acetate (8:2) to obtain dehydroabietic acid as colorless crystals after recrystallization in MeOH/H_2_O (164 g, 55% *w/w* yield). DHA was methylated with a solution of CH_2_N_2_ in ethyl ether to afford the corresponding dehydroabietic acid methyl ester (94%). Reduction of the methyl ester with LiAlH_4_ in dry tetrahydrofuran at reflux gave dehydroabietinol (78% yield).

#### 3.2.1. Preparation of Alkynyl Esters

Esterification of DHA and dehydroabietinol was performed using DCC/DMAP and appropriate alcohol (propargyl alcohol or 3-butyn-1-ol) or acid (4-pentynoic acid or 6-heptynoic acid) according to reference [18]. Briefly, DHA or alkynyl acid (1 eq) was dissolved in dry CH_2_Cl_2_ at room temperature under constant stirring. Then, DCC (1 eq) was added, followed by a catalytic amount of DMAP and alkynyl alcohol or dehydroabietinol (1 eq) dissolved in dry CH_2_Cl_2_. The reaction was stopped by adding H_2_O, extracted with CH_2_Cl_2_, dried over Na_2_SO_4_, concentrated and purified (60%–81% yield).

#### 3.2.2. General Procedure for the Synthesis of Triazole **1**–**16**

The alkynyl esters (1 eq) and the corresponding azide (1 eq) were dissolved in *t*-BuOH/H_2_O (1:1), followed by the addition of CuSO_4_·5H_2_O (2 mol%) and sodium ascorbate (10 mol%). The mixture was stirred at room temperature for 24 h. The reaction was stopped by adding H_2_O, extracted with CH_2_Cl_2_, dried over anhydrous Na_2_SO_4_, concentrated and purified by column chromatography on silica gel (54%–87% yield).

*18-((1-Phenyl-1H-1,2,3-triazol-4-yl)methyl)-8,11,13-abietatrienoate* (**1**). pale yellow resin; 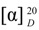
+27 (*c* 0.016, CHCl_3_); IR ν_max_ (film) 2928, 2870, 1720, 1461, 1245, 755 cm^−1^; ^1^H-NMR (CDCl_3_): δ 8.02 (1H, s, H-3'), 7.72 (2H, d, *J* = 7.8 Hz, H-2'' and H-6''), 7.54 (2H, t, *J* = 7.8 Hz, H-3'' and H-5''), 7.46 (1H, t, *J* = 7.3 Hz, H-4''), 7.14 (1H, d, *J* = 8.2 Hz, H-11), 6.98 (1H, dd, *J* = 8.2, 1.5 Hz, H-12), 6.84 (1H, d, *J* = 1.5 Hz, H-14), 5.30 (2H, s, H-1'), 2.88 (2H, m, H-7), 2.79 (1H, m, H-15), 2.28 (1H, brd, *J* = 12.7 Hz, H-1*β*), 2.24 (1H, dd, *J* = 12.2, 1.2 Hz, H-5), 1.28 (3H, s, H-19), 1.21 (6H, d, *J* = 6.9 Hz, H-16 and H-17), 1.20 (3H, s, H-20);^13^C-NMR (CDCl_3_): δ 178.96 (C-18), 147.15 (C-9), 146.13 (C-13), 144.93 (C-2'), 137.34 (C-1''), 135.02 (C-8), 130.21 (2C, C-3'' and C-5''), 129.34 (C-4''), 127.32 (C-14), 124.57 (C-11), 124.33 (C-12), 121.06 (2C, C-2'' and C-6''), 123.64 (C-3'), 58.12 (C-1'), 48.04 (C-4), 45.24 (C-5), 38.29, 37.34, 36.82, 33.85, 30.38, 25.57, 24.38 (2C, C-16 and C-17), 22.09, 18.92, 16.91; EIMS *m/z* 458.2462 [M+H]^+ ^(calcd for C_29_H_36_N_3_O_2_, 458.2807). 

*18-(2-(1-Phenyl-1H-1,2,3-triazol-4-yl)ethyl)-8,11,13-abietatrienoate* (**2**). colorless resin; 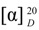
+17 (*c* 0.025, CHCl_3_); IR ν_max_ (film) 2930, 2865, 1726, 1470, 1245, 761 cm^−1^; ^1^H-NMR (CDCl_3_): δ 7.79 (1H, s, H-4'), 7.63 (2H, d, *J* = 7.6 Hz, H-2'' and H-6''), 7.50 (2H, t, *J* = 7.6 Hz, H-3'' and H-5''), 7.42 (1H, t, *J* = 7.3 Hz, H-4''), 7.16 (1H, d, *J* = 8.1 Hz, H-11), 6.99 (1H, brd, *J* = 8.2 Hz, H-12), 6.84 (1H, brs, H-14), 4.43 (2H, t, *J* = 6.6 Hz, H-1'), 3.15 (2H, t, *J* = 6.6 Hz, H-2'), 2.83 (2H, m, H-7), 2.71 (1H, m, H-15), 2.29 (1H, brd, *J* = 12.2 Hz, H-1*β*), 2.24 (1H, dd, *J* = 12.2, 1.2, H-5), 1.26 (3H, s, H-19), 1.21 (6H, d, *J* = 6.9 Hz, H-16 and H-17), 1.19 (3H, s, H-20); ^13^C-NMR (CDCl_3_): δ 178.35 (C-18), 146.73 (C-9), 145.68 (C-13), 144.90 (C-3'), 137.03 (C-1''), 134.49 (C-8), 129.71 (2C, C-3'' and C-5''), 128.56 (C-4''), 126.85 (C-14), 124.11 (C-11), 123.90 (C-12), 120.37 (2C, C-2'' and C-6''), 119.70 (C-4'), 63.10 (C-1'), 47.62 (C-4), 44.83 (C-5), 37.96, 36.89, 36.60, 33.38, 29.99, 25.59, 25.14, 23.92 (2C, C-16 and C-17), 21.70, 18.52, 16.50; EIMS *m/z* 472.2695 [M+H]^+ ^(calcd for C_30_H_38_N_3_O_2_, 472.2964). 

*18-((((1-Phenylthio)methyl)-1H-1,2,3-triazol-4-yl)methyl)-8,11,13-abietatrienoate* (**3**). pale yellow resin; 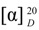
+33 (*c* 0.013, CHCl_3_); IR ν_max_ (film) 2922, 2852, 1714, 1442, 1238, 742 cm^−1^; ^1^H-NMR (CDCl_3_): δ 7.59 (1H, s, H-3'), 7.29 (5H, m, Ph), 7.11 (1H, d, *J* = 8.1 Hz, H-11), 6.96 (1H, brd, *J* = 8.1 Hz, H-12), 6.81 (1H, brs, H-14), 5.50 (2H, s, CH2S), 5.16 (2H, s, H-1'), 2.81 (2H, m, H-7), 2.72 (1H, m, H-15), 2.26 (1H, brd, *J* = 12.7 Hz, H-1*β*), 2.14 (1H, dd, *J* = 12.2, 1.5, Hz, H-5), 1.22 (3H, s, H-19), 1.17 (6H, d, *J* = 6.9 Hz, H-16 and H-17), 1.16 (3H, s, H-20); ^13^C-NMR (CDCl_3_): δ 178.90 (C-18), 147.05 (C-9), 146.12 (C-13), 143.92 (C-2'), 134.91 (C-8), 132.63 (2C, C-2'' and C-6''), 131.98 (C-1''), 129.88 (2C, C-3'' and C-5''), 129.18 (C-4''), 127.24 (C-14), 124.50 (C-11), 124.30 (C-12), 123.66 (C-3'), 57.93 (C-1'), 54.35 (CH_2_S), 47.96 (C-4), 45.18 (C-5), 38.25, 37.26, 36.78, 33.97, 30.31, 25.46, 24.29 (2C, C-16 and C-17), 22.03, 18.34, 16.77; EIMS *m/z* 504.2641 [M+H]^+^ (calcd for C_30_H_38_N_3_O_2_S, 504.2684). 

*18-(2-(((1-Phenylthio)methyl)-1H-1,2,3-triazol-4-yl)ethyl)-8,11,13-abietatrienoate* (**4**). pale yellow resin; 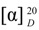
+14 (*c* 0.014, CHCl_3_); IR ν_max_ (film) 2928, 2867, 1722, 1440, 1242, 749 cm^−1^; ^1^H-NMR (CDCl_3_): δ 7.36 (1H, s, H-4'), 7.29 (5H, m, Ph), 7.14 (1H, d, *J* = 8.1 Hz, H-11), 6.99 (1H, brd, *J* = 8.1 Hz, H-12), 6.86 (1H, brs, H-14), 5.45 (2H, s, CH_2_S), 4.29 (2H, t, *J* = 6.6 Hz, H-1'), 3.03 (2H, t, *J* = 6.6 Hz, H-2'), 2.82 (2H, m, H-7), 2.70 (1H, m, H-15), 2.28 (1H, brd, *J* = 12.7 Hz, H-1*β*), 2.12 (1H, dd, *J* = 12.2, 1.5 Hz, H-5), 1.22 (6H, d, *J* = 6.9 Hz, H-16 and H-17), 1.21 (3H, s, H-19), 1.18 (3H, s, H-20); ^13^C-NMR (CDCl_3_): δ 178.24 (C-18), 146.77 (C-9), 145.74 (C-13), 144.81 (C-3'), 134.54 (C-8), 131.92 (C-1''), 131.79 (2C, C-2'' and C-6''), 129.24 (2C, C-3'' and C-5''), 128.50 (C-4''), 126.87 (C-14), 124.11 (C-11), 123.93 (C-12), 120.95 (C-4'), 63.15 (C-1'), 53.46 (CH_2_S), 47.55 (C-4), 44.82 (C-5), 37.96, 36.85, 36.50, 33.92, 29.91, 25.59, 25.08, 23.97 (2C, C-16 and C-17), 21.57, 18.49, 16.44; EIMS *m/z* 518.2164 [M+H]^+^ (calcd for C_31_H_40_N_3_O_2_S, 518.2841). 

*18-(3-(1-Phenyl-1H-1,2,3-triazol-4-yl)propanoyloxy)-8,11,13-abietatriene* (**5**). white resin; 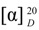
+20 (*c* 0.022, CHCl_3_); IR ν_max_ (film) 2925, 2864, 1726, 1463, 1246, 761 cm^−1^; ^1^H-NMR (CDCl_3_): δ 7.78 (1H, s, H-5'), 7.70 (2H, d, *J* = 7.6 Hz, H-2'' and H-6''), 7.53 (2H, t, *J* = 7.6 Hz, H-3'' and H-5''), 7.42 (1H, t, *J* = 7.3 Hz, H-4''), 7.16 (1H, d, *J* = 8.1 Hz, H-11), 6.99 (1H, brd, *J* = 8.1 Hz, H-12), 6.86 (1H, brs, H-14), 3.98 (1H, d, *J* = 10.9 Hz, H-18) 3.75 (1H, d, *J* = 10.9 Hz, H-18), 3.11 (2H, t, *J* = 7.3 Hz, H-3'), 2.86 (2H, m, H-7), 2.81 (1H, m, H-15), 2.79 (2H, t, *J* = 7.3 Hz, H-2'), 2.26 (1H, brd, *J* = 12.7 Hz, H-1*β*), 1.22 (6H, d,* J* = 6.9 Hz, H-16 and H-17), 1.20 (3H, s, H-20), 0.92 (3H, s, H-19); ^13^C-NMR (CDCl_3_): δ 173.30 (C-1'), 147.51 (C-4'), 147.46 (C-9), 146.02 (C-13), 137.54 (C-1''), 135.06 (C-8), 130.11 (2C, C-3'' and C-5''), 128.96 (C-4''), 127.28 (C-14), 124.71 (C-11), 124.32 (C-12), 120.82 (2C, C-2'' and C-6''), 119.91 (C-5'), 73.16 (C-18), 44.78 (C-5), 38.65, 37.82, 37.20, 35.96, 34.03, 33.84, 30.65, 25.78, 24.39 (2C, C-16 and C-17), 21.46, 19.42, 18.91, 17.84; EIMS *m/z* 486.3360 [M+H]^+^ (calcd for C_31_H_40_N_3_O_2_, 486.3120). 

*18-(5-(1-Phenyl-1H-1,2,3-triazol-4-yl)pentanoyloxy)-8,11,13-abietatriene* (**6**). white resin; 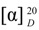
+25 (*c* 0.014, CHCl_3_); IR ν_max_ (film) 2930, 2864, 1726, 1461, 1248, 761 cm^−1^; ^1^H-NMR (CDCl_3_): δ 7.70 (1H, s, H-7'), 7.71 (2H, d, *J* = 7.4 Hz, H-2'' and H-6''), 7.53 (2H, t, *J* = 7.4 Hz, H-3'' and H-5''), 7.42 (1H, t, *J* = 7.4 Hz, H-4''), 7.18 (1H, d, *J* = 8.1 Hz, H-11), 6.99 (1H, brd, *J* = 8.1 Hz, H-12), 6.88 (1H, brs, H-14), 3.97 (1H, d, *J* = 10.9 Hz, H-18) 3.71 (1H, d, *J* = 10.9 Hz, H-18), 2.85 (2H, m, H-7), 2.83 (1H, m, H-15), 2.80 (2H, t, *J* = 7.3 Hz, H-5'), 2.36 (2H, t, *J* = 7.3 Hz, H-2'), 2.27 (1H, brd, *J* = 12.9 Hz, H-1*β*), 1.23 (6H, d, *J* = 6.9 Hz, H-16 and H-17), 1.21 (3H, s, H-20), 0.93 (3H, s, H-19); ^13^C-NMR (CDCl_3_): δ 174.13 (C-1'), 148.86 (C-6'), 147.54 (C-9), 146.03 (C-13), 137.66 (C-1''), 135.12 (C-8), 130.09 (2C, C-3'' and C-5''), 128.87 (C-4''), 127.29 (C-14), 124.71 (C-11), 124.31 (C-12), 120.83 (2C, C-2'' and C-6''), 119.33 (C-7'), 72.84 (C-18), 44.68 (C-5), 38.70, 37.84, 37.20, 35.99, 34.47, 33.83, 30.66, 29.19, 25.78, 25.74, 24.94, 24.38 (2C, C-16 and C-17), 19.39, 18.95, 17.91; EIMS *m/z* 514.3686 [M+H]^+^ (calcd for C_31_H_44_N_3_O_2_, 514.3433). 

*18-(3-(1-Benzyl-1H-1,2,3-triazol-4-yl)propanoyloxy)-8,11,13-abietatriene* (**7**). pale yellow resin; 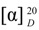
+16 (*c* 0.039, CHCl_3_); IR ν_max_ (film) 2934, 2867, 1726, 1458, 1240, 727 cm^−1^; ^1^H-NMR (CDCl_3_): δ 7.36 (1H, s, H-5'), 7.34 (2H, d, * J* = 7.6 Hz, H-2'' and H-6''), 7.24 (2H, t, *J* = 7.6 Hz, H-3'' and H-5''), 7.22 (1H, t, *J* = 7.3 Hz, H-4''), 7.17 (1H, d, *J* = 8.1 Hz, H-11), 6.99 (1H, brd, *J* = 8.1 Hz, H-12), 6.88 (1H, brs, H-14), 5.43 (2H, s, CH_2_Ph), 3.94 (1H, d, *J* = 10.9 Hz, H-18) 3.71 (1H, d, *J* = 10.9 Hz, H-18), 3.00 (2H, t, *J* = 7.3 Hz, H-3'), 2.85 (2H, m, H-7), 2.80 (1H, m, H-15), 2.70 (2H, t, *J* = 7.3 Hz, H-2'), 2.27 (1H, brd, *J* = 12.7 Hz, H-1*β*), 1.22 (6H, d, *J* = 6.9 Hz, H-16 and H-17), 1.21 (3H, s, H-20), 0.90 (3H, s, H-19); ^13^C-NMR (CDCl_3_): δ 173.24 (C-1'), 147.47 (C-9), 147.24 (C-4'), 146.02 (C-13), 135.24 (C-1''), 135.07 (C-8), 129.48 (2C, C-3'' and C-5''), 129.06 (C-4''), 128.40 (2C, C-2'' and C-6''), 127.29 (C-14), 124.71 (C-11), 124.33 (C-12), 121.44 (C-5'), 73.06 (C-18), 54.40 (CH_2_Ph), 44.77 (C-5), 38.66, 37.83, 37.19, 35.93, 34.04, 33.84, 30.65, 25.79, 24.40 (2C, C-16 and C-17), 21.51, 19.41, 18.92, 17.83; EIMS *m/z* 500.2639 [M+H]^+^ (calcd for C_32_H_42_N_3_O_2_, 500.3277). 

*18-(5-(1-Benzyl-1H-1,2,3-triazol-4-yl)pentanoyloxy)-8,11,13-abietatriene* (**8**). pale yellow resin; 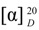
+10 (*c* 0.091, CHCl_3_); IR ν_max_ (film) 2934, 2867, 1729, 1458, 1220, 751 cm^−1^; ^1^H-NMR (CDCl_3_): δ 7.36 (1H, s, H-7'), 7.34 (2H, d, * J* = 7.6 Hz, H-2'' and H-6''), 7.25 (2H, t, *J* = 7.6 Hz, H-3'' and H-5''), 7.22 (1H, t, *J* = 7.3 Hz, H-4''), 7.19 (1H, d, *J* = 8.1 Hz, H-11), 7.00 (1H, brd, *J* = 8.1 Hz, H-12), 6.90 (1H, brs, H-14), 5.48 (2H, s, CH_2_Ph), 3.95 (1H, d, *J* = 10.9 Hz, H-18) 3.70 (1H, d, *J* = 10.9 Hz, H-18), 2.85 (2H, m, H-7), 2.80 (1H, m, H-15), 2.69 (2H, t, *J* = 7.3 Hz, H-5'), 2.32 (2H, t, *J* = 7.3 Hz, H-2'), 2.27 (1H, brd, *J* = 12.9 Hz, H-1*β*), 1.23 (6H, d, *J* = 6.9 Hz, H-16 and H-17), 1.22 (3H, s, H-20), 0.94 (3H, s, H-19); ^13^C-NMR (CDCl_3_): δ 174.13 (C-1'), 148.63 (C-6'), 147.55 (C-9), 146.02 (C-13), 135.36 (C-1''), 135.13 (C-8), 129.47 (2C, C-3'' and C-5''), 129.03 (C-4''), 128.37 (2C, C-2'' and C-6''), 127.30 (C-14), 124.73 (C-11), 124.32 (C-12), 121.04 (C-7'), 72.80 (C-18), 54.38 (CH_2_Ph), 44.68 (C-5), 38.71, 37.84, 37.19, 35.97, 34.45, 33.83, 30.67, 29.23, 25.81, 24.97, 24.41 (2C, C-16 and C-17), 19.39, 18.95, 17.91; EIMS *m/z* 528.4121 [M+H]^+^ (calcd for C_34_H_46_N_3_O_2_, 528.3590). 

*18-(3-(((1-Phenylthio)methyl)-1H-1,2,3-triazol-4-yl)propanoyloxy)-8,11,13-abietatriene* (**9**). yellow resin; 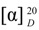
+7 (*c* 0.197, CHCl_3_); IR ν_max_ (film) 2931, 2864, 1726, 1442, 1250, 749 cm^−1^; ^1^H-NMR (CDCl_3_): δ 7.37 (1H, s, H-5'), 7.31 (5H, m, Ph), 7.20 (1H, d, *J* = 8.1 Hz, H-11), 7.02 (1H, brd, *J* = 8.1 Hz, H-12), 6.92 (1H, brs, H-14), 5.51 (2H, s, CH_2_S), 4.00 (1H, d, *J* = 10.9 Hz, H-18) 3.73 (1H, d, *J* = 10.9 Hz, H-18), 3.02 (2H, t, *J* = 7.3 Hz, H-3'), 2.87 (2H, m, H-7), 2.82 (1H, m, H-15), 2.72 (2H, t, *J* = 7.3 Hz, H-2'), 2.28 (1H, brd, *J* = 12.7 Hz, H-1*β*), 1.25 (6H, d, *J* = 6.9 Hz, H-16 and H-17), 1.23 (3H, s, H-20), 0.94 (3H, s, H-19); ^13^C-NMR (CDCl_3_): δ 173.11 (C-1'), 147.47 (C-9), 147.37 (C-4'), 146.04 (C-13), 135.08 (C-8), 132.50 (3C, C-1'', C-2'' and C-6''), 129.86 (2C, C-3'' and C-5''), 128.99 (C-4''), 127.31 (C-14), 124.74 (C-11), 124.36 (C-12), 121.09 (C-5'), 73.00 (C-18), 53.96 (CH_2_S), 44.72 (C-5), 38.68, 37.84, 37.23, 35.94, 33.97, 33.86, 30.67, 25.83, 24.44 (2C, C-16 and C-17), 21.49, 19.41, 18.94, 17.89; EIMS *m/z* 532.3104 [M+H]^+^ (calcd for C_32_H_42_N_3_O_2_S, 532.2997). 

*18-(5-(((1-Phenylthio)methyl)-1H-1,2,3-triazol-4-yl))pentanoyloxy)-8,11,13-abietatriene* (**10**). yellow resin; 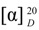
+11 (*c* 0.256, CHCl_3_); IR ν_max_ (film) 2937, 2867, 1725, 1467, 1251, 749 cm^−1^; ^1^H-NMR (CDCl_3_): δ 7.32 (5H, m, Ph), 7.28 (1H, s, H-7'), 7.21 (1H, d, *J* = 8.1 Hz, H-11), 7.03 (1H, brd, *J* = 8.1 Hz, H-12), 6.92 (1H, brs, H-14), 5.59 (2H, s, CH_2_S), 4.00 (1H, d, *J* = 10.9 Hz, H-18), 3.74 (1H, d, *J* = 10.9 Hz, H-18), 2.87 (2H, m, H-7), 2.85 (1H, m, H-15), 2.70 (2H, t, *J* = 7.3 Hz, H-5'), 2.34 (2H, t, *J* = 7.3 Hz, H-2'), 2.32 (1H, brd, *J* = 12.9 Hz, H-1*β*), 1.25 (6H, d, *J* = 6.9 Hz, H-16 and H-17), 1.24 (3H, s, H-20), 0.96 (3H, s, H-19); ^13^C-NMR (CDCl_3_): δ 174.08 (C-1'), 148.69 (C-6'), 147.54 (C-9), 146.02 (C-13), 135.12 (C-8), 132.64 (2C, C-2'' and C-6''), 132.48 (C-1''), 129.86 (2C, C-3'' and C-5''), 129.02 (C-4''), 127.31 (C-14), 124.74 (C-11), 124.34 (C-12), 120.71 (C-7'), 72.80 (C-18), 54.04 (CH_2_S), 44.68 (C-5), 38.72, 37.85, 37.21, 35.99, 34.29, 33.87, 30.69, 29.14, 25.82, 25.70, 24.85, 24.43 (2C, C-16 and C-17), 19.41, 18.97, 17.94; EIMS *m/z* 560.4263 [M+H]^+^ (calcd for C_34_H_46_N_3_O_2_S, 560.3310). 

*18-(3-(1-(4-Anisidyl)-1H-1,2,3-triazol-4-yl)propanoyloxy)-8,11,13-abietatriene* (**11**). white resin; 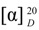
+21 (*c* 0.017, CHCl_3_); IR ν_max_ (film) 2928, 2864, 1726, 1442, 1257, 833 cm^−1^; ^1^H-NMR (CDCl_3_): δ 7.70 (1H, s, H-5'), 7.59 (2H, d, * J* = 9.0 Hz, H-2'' and H-6''), 7.16 (1H, d, *J* = 8.1 Hz, H-11), 7.00 (2H, d, *J* = 9.0 Hz, H-3'' and H-5''), 6.99 (1H, brd, *J* = 8.1 Hz, H-12), 6.86 (1H, brs, H-14), 3.98 (1H, d, *J* = 10.9 Hz, H-18), 3.86 (3H, s, PhOMe), 3.75 (1H, d, *J* = 10.9 Hz, H-18), 3.10 (2H, t,*J* = 7.3 Hz, H-3'), 2.87 (2H, m, H-7), 2.82 (1H, m, H-15), 2.78 (2H, t, * J* = 7.3 Hz, H-2'), 2.26 (1H, brd, *J* = 12.7 Hz, H-1*β*), 1.22 (6H, d, *J* = 6.9 Hz, H-16 and H-17), 1.20 (3H, s, H-20), 0.93 (3H, s, H-19); ^13^C-NMR (CDCl_3_): δ 173.32 (C-1'), 160.07 (C-4''), 147.46 (C-9), 147.29 (C-4'), 146.01 (C-13), 135.06 (C-8), 131.05 (C-1''), 127.28 (C-14), 124.71 (C-11), 124.32 (C-12), 122.45 (2C, C-2'' and C-6''), 120.09 (C-5'), 115.11 (2C, C-3'' and C-5''), 73.15 (C-18), 56.03 (OMe), 44.79 (C-5), 38.66, 37.83, 37.20, 35.96, 34.08, 33.84, 30.65, 25.78, 24.40 (2C, C-16 and C-17), 21.47, 19.42, 18.92, 17.84; EIMS *m/z* 516.4582 [M+H]^+^ (calcd for C_32_H_42_N_3_O_3_, 516.3226). 

*18-(5-(1-(4-Anisidyl)-1H-1,2,3-triazol-4-yl)pentanoyloxy)-8,11,13-abietatriene* (**12**). white resin; 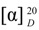
+12 (*c* 0.060, CHCl_3_); IR ν_max_ (film) 2937, 2867, 1729, 1461, 1257, 830 cm^−1^; ^1^H-NMR (CDCl_3_): δ 7.61 (1H, s, H-7'), 7.60 (2H, d, * J* = 8.9 Hz, H-2'' and H-6''), 7.17 (1H, d, *J* = 8.1 Hz, H-11), 7.00 (2H, d, *J* = 9.0 Hz, H-3'' and H-5''), 6.99 (1H, brd, *J* = 8.1 Hz, H-12), 6.88 (1H, brs, H-14), 3.97 (1H, d, *J* = 10.9 Hz, H-18), 3.86 (3H, s, PhOMe), 3.71 (1H, d, *J* = 10.9 Hz, H-18), 2.86 (2H, m, H-7), 2.81 (1H, m, H-15), 2.78 (2H, t, *J* = 7.3 Hz, H-5'), 2.35 (2H, t, *J* = 7.3 Hz, H-2'), 2.27 (1H, brd, *J* = 12.9 Hz, H-1*β*), 1.22 (6H, d, *J* = 6.9 Hz, H-16 and H-17), 1.21 (3H, s, H-20), 0.93 (3H, s, H-19); ^13^C-NMR (CDCl_3_): δ 174.15 (C-1'), 160.01 (C-4''), 148.66 (C-6'), 147.54 (C-9), 146.02 (C-13), 135.12 (C-8), 131.17 (C-1''), 127.29 (C-14), 124.71 (C-11), 124.30 (C-12), 122.49 (2C, C-2'' and C-6''), 119.54 (C-7'), 115.10 (2C, C-3'' and C-5''), 72.84 (C-18), 56.03 (OMe), 44.68 (C-5), 38.69, 37.83, 37.19, 35.99, 34.47, 33.83, 30.66, 29.23, 25.79, 25.74, 24.95, 24.38 (2C, C-16 and C-17), 19.39, 18.94, 17.90; EIMS *m/z* 544.4256 [M+H]^+^ (calcd for C_34_H_46_N_3_O_3_, 544.3539). 

*18-(3-(1-(3-Chlorophenyl)-1H-1,2,3-triazol-4-yl)propanoyloxy)-8,11,13-abietatriene* (**13**). white resin; 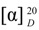
+19 (*c* 0.014, CHCl_3_); IR ν_max_ (film) 2928, 2864, 1726, 1460, 1248, 776 cm^−1^; ^1^H-NMR (CDCl_3_): δ 7.78 (1H, s, H-5'), 7.76 (1H, s, H-2''), 7.61 (1H, d, *J* = 8.0 Hz, H-6''), 7.45 (1H, t, *J* = 8.0 Hz, H-5''), 7.39 (1H, d, *J* = 8.0 Hz, H-4''), 7.16 (1H, d, *J* = 8.1 Hz, H-11), 6.99 (1H, brd, *J* = 8.1 Hz, H-12), 6.86 (1H, brs, H-14), 3.98 (1H, d, *J* = 10.9 Hz, H-18) 3.75 (1H, d, *J* = 10.9 Hz, H-18), 3.11 (2H, t, *J* = 7.3 Hz, H-3'), 2.86 (2H, m, H-7), 2.80 (1H, m, H-15), 2.78 (2H, t, *J* = 7.3 Hz, H-2'), 2.26 (1H, brd, *J* = 12.7 Hz, H-1*β*), 1.22 (6H, d, *J* = 6.9 Hz, H-16 and H-17), 1.20 (3H, s, H-20), 0.92 (3H, s, H-19); ^13^C-NMR (CDCl_3_): δ 173.23 (C-1'), 147.81 (C-4'), 147.45 (C-9), 146.03 (C-13), 138.36 (C-3''), 135.94 (C-1''), 135.04 (C-8), 131.20 (C-5''), 129.00 (C-4''), 127.28 (C-14), 124.71 (C-11), 124.33 (C-12), 121.02 (C-2''), 119.85 (C-5'), 118.72 (C-6''), 73.15 (C-18), 44.74 (C-5), 38.64, 37.82, 37.21, 35.96, 33.90, 33.84, 30.64, 25.77, 24.40 (2C, C-16 and C-17), 21.40, 19.41, 18.91, 17.84; EIMS *m/z* 520.2411 [M+H]^+^ (calcd for C_31_H_39_ClN_3_O_2_, 520.2731). 

*18-(5-(1-(3-Chlorophenyl)-1H-1,2,3-triazol-4-yl)pentanoyloxy)-8,11,13-abietatriene* (**14**). pale yellow resin; 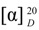
+17 (*c* 0.012, CHCl_3_); IR ν_max_ (film) 2934, 2864, 1726, 1461, 1241, 782 cm^−1^; ^1^H-NMR (CDCl_3_): δ 7.76 (1H, s, H-2''), 7.69 (1H, s, H-5'), 7.63 (1H, d, *J* = 8.0 Hz, H-6''), 7.45 (1H, t, *J* = 8.0 Hz, H-5''), 7.39 (1H, d, *J* = 8.0 Hz, H-4''), 7.17 (1H, d, *J* = 8.1 Hz, H-11), 6.99 (1H, brd, *J* = 8.1 Hz, H-12), 6.88 (1H, brs, H-14), 3.97 (1H, d, * J* = 10.9 Hz, H-18) 3.72 (1H, d, *J* = 10.9 Hz, H-18), 2.86 (2H, m, H-7), 2.83 (1H, m, H-15), 2.80 (2H, t, *J* = 7.3 Hz, H-5'), 2.35 (2H, t, *J* = 7.3 Hz, H-2'), 2.27 (1H, brd, *J* = 12.9 Hz, H-1*β*), 1.22 (6H, d, *J* = 6.9 Hz, H-16 and H-17), 1.20 (3H, s, H-20), 0.94 (3H, s, H-19); ^13^C-NMR (CDCl_3_): δ 174.06 (C-1'), 149.16 (C-6'), 147.55 (C-9), 146.04 (C-13), 138.48 (C-3''), 135.91 (C-1''), 135.10 (C-8), 131.16 (C-5''), 128.90 (C-4''), 127.28 (C-14), 124.70 (C-11), 124.30 (C-12), 121.03 (C-2''), 119.19 (C-7'), 118.75 (C-6''), 72.83 (C-18), 44.70 (C-5), 38.73, 37.84, 37.21, 36.00, 34.43, 33.83, 30.65, 29.10, 25.75, 25.68, 24.90, 24.36 (2C, C-16 and C-17), 19.40, 18.94, 17.90; EIMS *m/z* 548.4267 [M+H]^+^ (calcd for C_33_H_43_ClN_3_O_2_, 548.3044). 

*18-(3-(1-(4-Toluenesulfonyl)-1H-1,2,3-triazol-4-yl)propanoyloxy)-8,11,13-abietatriene* (**15**). pale yellow resin; 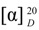
+22 (*c* 0.022, CHCl_3_); IR ν_max_ (film) 2934, 2864, 1729, 1461, 1240, 816 cm^−1^; ^1^H-NMR (CDCl_3_): δ 7.97 (2H, d, *J* = 8.1 Hz, H-2'' and H-6''), 7.91 (1H, s, H-5'), 7.37 (2H, d, *J* = 8.1 Hz, H-3'' and H-5''), 7.17 (1H, d, *J* = 8.1 Hz, H-11), 6.99 (1H, brd, *J* = 8.1 Hz, H-12), 6.88 (1H, brs, H-14), 3.94 (1H, d, *J* = 10.9 Hz, H-18) 3.72 (1H, d, * J* = 10.9 Hz, H-18), 3.02 (2H, t, *J* = 7.3 Hz, H-3'), 2.88 (2H, m, H-7), 2.81 (1H, m, H-15), 2.69 (2H, t, *J* = 7.3 Hz, H-2'), 2.43 (3H, s, PhMe), 2.27 (1H, brd, *J* = 12.7 Hz, H-1*β*), 1.22 (6H, d, *J* = 6.9 Hz, H-16 and H-17), 1.20 (3H, s, H-20), 0.90 (3H, s, H-19); ^13^C-NMR (CDCl_3_): δ 172.82 (C-1'), 147.59 (C-4'), 147.44 (C-9), 146.64 (C-4''), 146.04 (C-13), 135.04 (C-8), 133.59 (C-1''), 130.82 (2C, C-2'' and C-6''), 129.05 (2C, C-3'' and C-5''), 127.30 (C-14), 124.68 (C-11), 124.33 (C-12), 121.43 (C-5'), 73.25 (C-18), 44.76 (C-5), 38.65, 37.81, 37.17, 35.95, 33.84, 33.50, 30.62, 25.77, 24.39 (2C, C-16 and C-17), 21.18, 19.40, 18.89, 17.80; EIMS *m/z* 564.3065 [M+H]^+^ (calcd for C_32_H_42_N_3_O_4_S, 564.2896). 

*18-(5-(1-(4-Toluenesulfonyl)-1H-1,2,3-triazol-4-yl)pentanoyloxy)-8,11,13-abietatriene* (**16**). pale yellow resin; 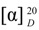
+30 (*c* 0.035, CHCl_3_); IR ν_max_ (film) 2937, 2867, 1726, 1451, 1250, 816 cm^−1^; ^1^H-NMR (CDCl_3_): δ 7.93 (2H, d, *J* = 8.1 Hz, H-2'' and H-6''), 7.72 (1H, s, H-5'), 7.32 (2H, d, *J* = 8.1 Hz, H-3'' and H-5''), 7.18 (1H, d, *J* = 8.1 Hz, H-11), 7.01 (1H, brd, *J* = 8.1 Hz, H-12), 6.89 (1H, brs, H-14), 3.99 (1H, d, *J* = 10.9 Hz, H-18) 3.66 (1H, d, * J* = 10.9 Hz, H-18), 2.86 (2H, m, H-7), 2.83 (1H, m, H-15), 2.81 (2H, t,*J* = 7.3 Hz, H-5'), 2.43 (3H, s, PhMe), 2.24 (2H, t, *J* = 7.3 Hz, H-2'), 2.20 (1H, brd, *J* = 12.9 Hz, H-1*β*), 1.22 (6H, d, *J* = 6.9 Hz, H-16 and H-17), 1.21 (3H, s, H-20), 0.93 (3H, s, H-19); ^13^C-NMR (CDCl_3_): δ 174.20 (C-1'), 148.65 (C-6'), 147.60 (C-9), 146.10 (C-13), 145.42 (C-4''), 135.18 (C-8), 133.20 (C-1''), 129.98 (2C, C-2'' and C-6''), 128.76 (2C, C-3'' and C-5''), 127.33 (C-14), 124.72 (C-11), 124.33 (C-12), 119.33 (C-7'), 72.73 (C-18), 44.58 (C-5), 38.74, 37.83, 37.23, 35.94, 34.38, 33.83, 30.64, 28.63, 25.77, 25.70, 24.82, 24.40 (2C, C-16 and C-17), 22.08, 19.36, 18.92, 17.90; EIMS *m/z* 592.3027 [M+H]^+^ (calcd for C_34_H_46_N_3_O_4_S, 592.3209). 

### 3.3. Antiproliferative Assay

All human cell lines used in this work were purchased from the American Type Culture Collection (ATCC, Manasas, VA, USA). Normal lung MRC-5 fibroblasts (CCL-171), SK-MES-1 lung cancer cells (HTB-58) and J82 bladder carcinoma cells (HTB-1) were grown as monolayers in minimum essential Eagle medium (MEM) with Earles’s salts, 2 mM L-glutamine and 1.5 g/L sodium bicarbonate. Gastric adenocarcinoma AGS cells (CRL-1739) were grown as monolayers in Ham F-12 medium containing 1 mM L-glutamine and 1.5 g/L sodium bicarbonate. All media were supplemented with 10% heat-inactivated FBS, 100 IU/mL penicillin and 100 µg/mL streptomycin. Cells were grown in a humidified incubator with 5% CO_2_ in air at 37 °C. For the antiproliferative assay, cells were plated at a density of 5 × 10^4^ cells/mL. Cells were seeded in 96-well plates (100 µL/well). One day after seeding, cells were treated with medium containing the compounds at concentrations ranging from 0 up to 100 µM during 3 days. The compounds were dissolved in DMSO (1% final concentration) and complete medium. Untreated cells (medium containing 1% DMSO) were used as 100% viability controls. Etoposide (98% purity, Sigma-Aldrich, St. Louis, MO, USA) was used as reference compound. Each concentration was tested in sextuplicate and experiments were repeated 2 times. Cell viability was determined by means of the MTT reduction assay at the end of the incubation with the products. The results were transformed to percentage of controls and the IC_50_ value was obtained adjusting the dose-response curve to a sigmoidal model. The software used was OriginPro 8.1 [23].

## 4. Conclusions

Sixteen dehydroabietic acid trizaole derivatives were prepared using click chemistry. The derivatives contain structural differences such as variability of aromatic rings and linkers which allow performing structure–activity relationship. The compounds were assessed as antiproliferative agents in three human tumor cell lines and on normal fibroblasts. The most remarkable difference was observed for compounds **5** and **6**, differing in the length of the linker (n:1* vs.* n:3), as while compound **5** was the most active compound in this study, compound **6** was inactive. On the other hand, the tumor cell selectivity of the compounds **7** and **8** is very interesting.
